# The cross-talk between T and B lymphocytes and the oral microbiome in health and disease: a narrative review

**DOI:** 10.3389/fimmu.2026.1862609

**Published:** 2026-09-30

**Authors:** Iwona Ordyniec-Kwaśnica, Bartosz Słomiński, Mateusz Lampkowski, Anna Kudra, Damian Muszyński

**Affiliations:** 1 Division of Dental Prosthetics, Medical University of Gdańsk, Gdansk, Poland; 2 Department of Medical Immunology, Medical University of Gdańsk, Gdańsk, Poland; 3 ”Zdrowie“ LLC DENTUS Dental Clinics in Gdynia, Gdynia, Poland; 4 Pomeranian Dental Center Kryspin-Dent, Gdansk, Poland

**Keywords:** B lymphocytes, immunology, oral immune, oral microbiome, T lymphocytes

## Abstract

T and B lymphocytes are key components of the adaptive immune system, playing a central role in maintaining host homeostasis and protecting against a wide range of pathogens. A comparable form of homeostatic regulation can be observed in microbial ecosystems, including the oral cavity. This environment is extremely unstable and can be easily disrupted by various factors or stimuli. This review article presents the different types of T and B lymphocytes and their role in the human body, and attempts to answer the question of how these two basic types of immune system cells can affect the imbalance of the oral microbiome.

## 1 Introduction

In the oral cavity, adaptive immunity operates at a highly specialized mucosal interface that is continuously exposed to complex polymicrobial biofilms, dietary and environmental antigens, and recurrent mechanical perturbation (1, 2). Oral adaptive immunity must maintain immunological homeostasis in the presence of a dense and continuously evolving microbial community while retaining the capacity to mount protective responses when microbial dysbiosis or tissue perturbation occurs (3, 4). Adaptive immune responses, particularly those involving specific T-cell subsets and B-cell responses, interact with innate and stromal immune pathways to influence whether oral tissue homeostasis is maintained or chronic inflammation and alveolar bone loss develop (5, 6).

Changes in immune status may lead to alterations in microbiome composition and function. This manifests itself, for example, in the gastrointestinal tract where the local immune system is able to develop immune tolerance to commensal microbiota highlighting that the gastrointestinal tract harbors the body’s largest microbial biomass, dominated by billions of obligate anaerobes with cell densities exceeding 10^11^ per gram (7). In contrast, the oral cavity harbors one of the most diverse microbial communities in the human body displaying a distinct profile in species abundance and composition despite a lower total biomass (1). While the gut is heavily dominated by *Bacteroidetes* and *Firmicutes*, the oral microbiome exhibits a highly specific abundance of facultative anaerobes and distinctive genera such as *Streptococcus*, *Actinomyces*, and *Corynebacterium*, structurally organized across distinct mucosal and dental niches. (8–10).

Importantly, this compartmentalization is bridged by the “oral-gut axis,” through which billions of oral bacteria are translocated daily to the digestive tract. Under dysbiotic conditions, this continuum drives oral microbiota-driven immune modulation along the oral-gut axis, where translocated oral pathobionts bypass the gastric barrier to promote local gut inflammation and systemic immune signaling (11). This cross-talk directly shapes systemic “immune competence” the functional resilience of the immune system—which demonstrates tightly coordinated relationships with both the oral and gut microbiome compositions and directly impacts the activation profiles of circulating and tissue-resident lymphocyte populations (12). Consequently, expanding our focus to these interconnected microbial compartments is essential for a comprehensive understanding of how T- and B-cell responses are modulated in both local oral health and systemic inflammatory diseases (9).

Importantly, this relationship can also work in the opposite direction—elements of the microbiome can also modulate immune cell function (such as T and B cells). This interplay is further supported by the observation that immune system maturation occurs in parallel with the establishment of the microbiota during early life (4, 13). Moreover, evidence from experimental models indicates that the maternal microbiome can influence immune development in offspring (13). Beyond these interactions, the microbiome contributes to the maintenance of host homeostasis (4). Collectively, these findings suggest that the microbiome may be considered an integral component of the host immune system. These findings suggest that the microbiome is increasingly recognized as a major regulator of immune system development and function.

According to current knowledge, occurrence of immune-microbial homeostasis as well as development of dysbiosis or inflammatory disease may be a outcome of bidirectional interactions between oral microbial communities and T- and B-lymphocyte subsets. In particular, the balance between T helper 17 (Th17) and regulatory T (Treg) cells, mucosal-associated invariant T (MAIT)-cell activation, B-cell-mediated antibody production, have been presented to possibly take part in these processes. However, the available evidence remains fragmented and occasionally inconsistent because of substantial differences in study populations, experimental models, oral sampling sites, immune-cell phenotyping methods, and microbiome analysis techniques. Relatively few studies have simultaneously characterized oral microbial communities and detailed lymphocyte phenotypes within the same analytical framework. Therefore, this narrative review aims to summarize current knowledge on how T and B lymphocytes interact with the oral microbiome in health and dysbiosis, identify the main gaps and limitations in the available research, and explain the importance of these interactions in oral diseases. A complex understanding of these bidirectional interactions might be beneficial in the future development of immune-informed biomarkers and host-directed preventative and therapeutic treatments in dentistry.

## 2 Methods

### 2.1 Review design

This study was conducted as a narrative literature review aimed at providing a comprehensive and integrative overview of current knowledge regarding interactions between T and B lymphocytes and the oral microbiome in health and disease. The review was intended to identify major immunological pathways, summarize evidence from experimental and clinical studies, and highlight conceptual and methodological gaps in the field.

### 2.2 Literature search strategy

A focused literature search was performed using the PubMed and Google Scholar. PubMed was used as the primary biomedical database, whereas Google Scholar was used as a supplementary source to capture broader interdisciplinary literature and to support citation tracking. The following keywords and combinations of terms were used: “*T lymphocytes*”, “*T cells*”, “*B lymphocytes*”, “*B cells*”, “*oral microbiome*”, *“oral microbiota”, “dental plaque”, “homeostasis”, “dysbiosis”, “periodontitis”, “dental caries”*, and *“oral candidiasis*”.

Publications in English or Polish published between January 1985 and June 2026 were considered. This time frame was chosen to include foundational studies on T- and B-lymphocyte biology as well as more recent research on oral microbiome analysis and immune–microbial interactions. Original research articles, clinical and experimental studies, and review papers relevant to the topic were used to develop a comprehensive narrative synthesis.

### 2.3 Study selection and synthesis

The literature was organized thematically around T-cell responses, B-cell responses, oral immune–microbial homeostasis, and immune alterations associated with oral dysbiosis. Particular attention was given to evidence concerning the Th17/Treg balance, MAIT-cell responses, B-cell-mediated antibody production, and the role of immune–microbial interactions in periodontal disease, dental caries, and oral candidiasis.

## 3 Composition and role of the healthy oral microbiome

According to the definition provided in the review by Rajasekaran et al., the oral microbiome is a complex ecosystem of bacteria, fungi, archaea, and viruses that contribute to oral health. This system is extremely sensitive to external environmental factors such as tobacco, alcohol, lifestyle, and diet (2). All kinds of changes lead to various pathological conditions. That is why it is so important to maintain a healthy oral microbiome (2). Both Gram-positive and Gram-negative bacteria inhabit the oral cavity. Common Gram-positive genera include *Streptococcus, Peptostreptococcus, Actinomyces, Bifidobacterium, Corynebacterium*, and *Lactobacillus*, whereas representative Gram-negative genera *include Neisseria, Veillonella, Fusobacterium, Prevotella*, and *Porphyromonas* (14, 15).

Peptostreptococci are obligate anaerobic cocci commonly found within the oral microbiota. In a study by Rams et al. including more than 900 participants with different stages of periodontal disease, *Parvimonas micra* was identified among the associated bacterial taxa. The study demonstrated an association between the presence of *P. micra* and periodontal inflammation (16).

Streptococci are known as the primary colonizers of the oral cavity. Their function is dual, similar to that of the aforementioned *Peptostreptococcus*—on the one hand, they participate in shaping the oral microbiota and the immune system, and on the other hand, some of their representatives are cariogenic bacteria (17). The presence of streptococci is particularly noticeable on the surfaces of teeth and the tongue. Representatives of cariogenic bacteria belonging to streptococci include *S. mutans* and *S. sobrinus* (17). Their deleterious effects on dental tissues are associated with the capacity to produce glucans from sucrose, ferment dietary carbohydrates into organic acids, and survive in acidified microenvironments (18).

*Actinomyces* are non-spore-forming bacilli commonly associated with oral pathologies such as dental caries and periodontitis (19). *Actinomyces*, like *Streptococcus*, are classified as representatives of bacterial groups that appear relatively early in dental plaque (19). Cavalcanti et al. investigated the relationships between Streptococcus, Actinomyces, and Candida species, demonstrating their ability to form dual-species and polymicrobial biofilms. Interestingly, *A. oris* (a representative of *Actinomyces*) biofilm formation is markedly impaired in the absence of streptococcal partners (20).

*Bifidobacterium* is a genus of bacteria frequently studied for its probiotic properties. Its representatives, such as *B. animalis*, play an important role in bacterial co-aggregation and also have the ability to modulate the immune response. *B. lactis*, on the other hand, may have therapeutic effects in cases of periodontitis and inflammation of the mucous membrane around implants (21). Members of the genus *Bifidobacterium* occur naturally in the oral microbiota, including species such as *B. dentium* and *B. longum* (22).

*Corynebacterium* spp. have been described as important contributors to extracellular matrix production and biofilm formation.The most frequently detected species include *C. durum* and *C. matruchotii*, which have been associated with oral health–related biofilm organization (23). Their beneficial effects may be related to inhibitory interactions between *C. durum* and *C. matruchotii* and key oral pathogens such as *Candida albicans and Streptococcus mutans*, as reported by Treerat et al. This interference is proposed to occur through molecular-level mechanisms affecting gene expression and interspecies signaling (24).

*Lactobacillus* spp. are Gram-positive rod-shaped bacteria whose involvement in the development of carious lesions has been well documented. Several species within this genus have been associated with cariogenic lesions, including *L. fermentum* and *L. gasseri* (25). However, members of this genus may also exhibit probiotic properties. Lopes et al. conducted a comprehensive review of databases regarding commonly used probiotics in caries prevention and reported *L. rhamnosus* as one of the most frequently cited strains. This suggests that different strains within the same species or genus may exert divergent effects on caries development, acting either as cariogenic or beneficial microorganisms depending on context (26).

## 4 Adaptive immune cell subsets in oral mucosal immunity

The adaptive immune system of the oral mucosa does not function merely as a passive observer, but rather through dynamic, bidirectional communication (bidirectional cross-talk) with the local microbiome. Subpopulations of adaptive immune cells—including diverse T and B lymphocyte lineages—continuously integrate signals from commensal and pathogenic microorganisms to maintain tissue homeostasis and limit destructive inflammation.

### 4.1 T-Lymphocyte subsets and functional diversity

CD4^+^ helper T cells (Th cells) represent a heterogeneous population of immune cells that coordinate adaptive immune responses. Their activation is initiated by recognition of peptide antigens presented in the context of major histocompatibility complex class II (MHC II) molecules on antigen-presenting cells, in conjunction with co-stimulatory signals. This process triggers intracellular signaling pathways that lead to the differentiation of naive CD4^+^ T cells into distinct functional subsets, including Th1, Th2, Th17, T follicular helper (Tfh), and regulatory T (Treg) cells (27–29).

At the oral mucosal barrier, T-cell subsets function within a continuous bidirectional feedback loop where microbial signals direct lymphocyte differentiation, and T-cell cytokines dynamically regulate bacterial containment (5, 11, 30). This integrative cross-talk determines whether local T-cell responses preserve tissue homeostasis or promote microbial dysbiosis in diseased contexts (3, 6).

In healthy human gingiva, T cells constitute the predominant lymphocyte population within a broader immune network that includes abundant neutrophils, professional antigen-presenting cells, innate lymphoid cells, and relatively few B cells (30). CD4^+^ T cells comprise functionally specialized subsets, including Th1, Th2, Th17, Tfh, and regulatory T cells, whose distinct cytokine profiles and interactions with other immune and stromal cells contribute to gingival homeostasis (31).

Naive CD4^+^ T cells differentiate into distinct functional helper T-cell subsets with specialized roles in oral mucosal immunity. Among these, Th17 cells are crucial for antimicrobial defense against *Candida albicans* and extracellular pathogens, although their dysregulated expansion in response to dysbiotic biofilms promotes excessive neutrophil accumulation, osteoclast activation, and alveolar bone loss (5, 31, 32). Meanwhile, Th1 cells primarily produce interferon-γ (IFN-γ) to target intracellular pathogens, whereas Th2 cells secrete cytokines such as interleukin-4 (IL-4), interleukin-5 (IL-5), and interleukin-13 (IL-13) to support humoral immunity and tissue repair (33, 34). Furthermore, T follicular helper (Tfh) cells promote B-cell activation and antibody maturation via interleukin-21 (IL-21) and CD40L–CD40 interactions (35). In contrast, regulatory T (Treg) cells suppress excessive local inflammation and maintain immune tolerance to commensal biofilms, highlighting the Th17/Treg balance as a critical determinant of oral tissue homeostasis versus destruction (34). Although bidirectional interactions between the oral microbiome and specific T-cell populations, particularly Th17 and tissue-resident γδ T cells, are increasingly recognized, evidence directly linking individual Th1, Th2, Tfh, or Treg populations to defined changes in oral microbial community composition remains comparatively limited.

Both CD4^+^ and CD8^+^ T-cell populations are present in healthy human gingiva, contributing to tissue homeostasis. Upon antigenic stimulation, naive CD8^+^ T cells differentiate into cytotoxic effector T lymphocytes (CTLs) that eliminate target cells through perforin and granzyme secretion as well as Fas–FasL-mediated apoptosis (30, 34, 36). Wnt signaling can restrain terminal differentiation of CD8^+^ T cells and promote the generation of memory precursor cells, thereby supporting the formation of CD8^+^ memory T cells (37). Following the peak of the immune response, approximately 90–95% of CD8^+^ effector T cells undergo apoptosis during the contraction phase, while the remaining cells persist and differentiate into memory subsets. These include central memory T cells (T_CM_) and effector memory T cells (T_EM_), which differ in their homing properties and effector functions. In chronic oral mucosal lesions, such as Oral Lichen Planus (OLP), cytotoxic CD8^+^ T cells preferentially target basal keratinocytes, inducing basement membrane disruption and cell apoptosis. Under persistent antigenic stimulation, chronic inflammatory environments favor the accumulation of CD8^+^ TEMRA cells, which exhibit features of cellular senescence and a senescence-associated secretory phenotype (SASP), thereby contributing to sustained tissue inflammation and damage (38–42). Memory CD8^+^ T cells remain in a relatively quiescent state but can rapidly respond upon antigen re-exposure by proliferating and differentiating into effector cells (36).

Although these subsets may contribute to mucosal immune regulation, direct evidence linking some of them, particularly Th9, Tfh, and CD8^+^ T-cell subsets, to specific oral microbiome shifts remains limited. For this reason, their relevance to oral dysbiosis should be interpreted with caution and mainly in the context of broader immune regulation.

It is critical to distinguish observational associations from causal relationships when evaluating T-cell responses in oral diseases. While clinical cross-sectional studies demonstrate distinct shifts in local CD4^+^ and CD8^+^ profiles during oral dysbiosis, they primarily establish correlation. Mechanistic insights showing direct immune-driven microbial alterations or pathogen-induced T-cell polarization rely heavily on *in vitro* and animal models (such as mouse periodontitis models). Although these translational models provide valuable proof-of-concept, their clinical extrapolations must be interpreted cautiously given species-specific differences in mucosal biology and microbial composition.

### 4.2 Innate-like and unconventional lymphocyte populations

In recent years, the classification of T-lymphocyte subsets has expanded significantly beyond the traditional lineages. The transcription factor T-bet, while classically defining Th1 cells, is recognized as an important regulator of T-cell plasticity, including Treg and Th17 cell reprogramming in chronic inflammatory conditions (43). Furthermore, chronic antigenic stimulation and immunosenescence promote the accumulation of TEMRA cells (effector memory T cells re-expressing CD45RA), which exhibit terminal differentiation and a potent pro-inflammatory secretory profile that may contribute to chronic inflammation and tissue damage (42). Another newly identified subset, T peripheral helper (Tph) cells (CD4+CXCR5-PD-1^high^), has been shown to migrate directly into inflamed peripheral tissues rather than lymphoid organs, locally promoting B-cell differentiation and antibody production in chronic inflammatory diseases (44). In addition to conventional T cells, mucosal-associated invariant T (MAIT) cells— characterized by a semi-invariant TRAV1-2 (Vα7.2)-containing TCRα chain -play a pivotal role at the oral interface by recognizing microbial vitamin B metabolites via MR1, providing a rapid cytokine response (IFN-γ, IL-17) upon oral dysbiotic stimulation (45, 46).

Innate lymphoid cells (ILCs), although lacking rearranged antigen-specific receptors, are tissue-resident lymphocytes that respond rapidly to epithelial and myeloid signals at mucosal barriers. All major ILC subsets have been identified within oral and periodontal tissues. In human gingivitis and periodontitis samples, ILC1s were the predominant subset, and a proportion of these cells expressed receptor activator of nuclear factor κB ligand (RANKL), suggesting their potential involvement in osteoclast activation and periodontal bone resorption (47). Moreover, ILC1s and ILC3s, particularly NKp44^+^ ILC3s, were increased in the periodontal ligament of patients with periodontitis and demonstrated enhanced production of interferon-γ (IFN-γ) and interleukin-17A (IL-17A), indicating that these populations may contribute to local inflammatory responses (48). Experimental evidence concerning their contribution to oral candidiasis is more heterogeneous. One murine study identified IL-23-dependent, IL-17-producing ILCs as an important early source of antifungal cytokines, whereas another study attributed early oral IL-17 production predominantly to γδ T cells and natural Th17 cells rather than TCR-negative ILCs (49, 50). These findings indicate that the contribution of ILCs to oral barrier defense may depend on the experimental model, immune status, and type of microbial challenge.

Gamma delta T cells (γδ T cells) are unconventional T lymphocytes that combine features of innate and adaptive immunity and provide rapid responses at epithelial barriers. In murine gingiva, tissue-resident Vγ6^+^ γδ T cells were located preferentially within the epithelium adjacent to the dental biofilm and constituted a major source of IL-17. Their composition and activation were influenced by the local microbiota, whereas their experimental depletion increased gingival inflammation and altered oral microbial diversity, supporting a reciprocal relationship between γδ T cells and the oral microbiome (51). Gingival γδ T cells also produce amphiregulin, which supports tissue repair and limits periodontal pathology; loss of these cells increased disease severity in experimental periodontitis (52). In addition, oral-resident γδ T cells rapidly produced IL-17 during experimental oropharyngeal candidiasis, contributing to early antifungal defense together with natural Th17 cells (50). Collectively, these findings identify γδ T cells as important regulators of oral barrier integrity, epithelial repair, microbial control, and inflammatory homeostasis. However, most mechanistic evidence derives from murine models, and further studies are required to establish the corresponding functions of these cells in human oral mucosa.

### 4.3 B-lymphocyte subsets and mucosal humoral immunity

B lymphocytes represent a multifaceted cellular network at the oral interface, moving beyond classic systemic antibody production to engage in local immune–microbial regulation (5, 30). The functional state of mucosal B cells is dynamically tuned by local microbial exposure; in turn, B-cell activities—ranging from secretory immunoglobulin production to cytokine release—directly modulate the oral microbial ecosystem (53, 54). Evaluating mucosal B-cell lineages within this reciprocal relationship clarifies their dual capacity to preserve physiological microbial balance or contribute to local tissue pathology under persistent dysbiotic stimulation (55, 56).

B lymphocytes enable the recognition of specific antigenic epitopes through clonally diverse immunoglobulin (Ig) receptors expressed on their cell surface. As a component of the adaptive immune system, B lymphocytes perform multiple functions and engage in complex interactions with both innate and adaptive immune mechanisms.

In the oral environment, these B-cell functions are directly relevant to microbial recognition and mucosal immune defense. B-cell-mediated antibody production, particularly IgA secretion, may influence microbial adhesion, colonization, and the maintenance of oral microbial homeostasis. However, under dysbiotic conditions, chronic stimulation by periodontal pathogens may shift B-cell responses toward a more inflammatory profile, including mechanisms associated with RANKL expression and alveolar bone resorption.

#### 4.3.1 Functions of B lymphocytes

Studies have shown that interleukin-10 (IL-10) produced by B cells can attenuate or limit the progression of autoimmune and inflammatory diseases, highlighting the regulatory and immunosuppressive function of B cells (53).

In the local periodontal tissue microenvironment, B cells engage in bidirectional interactions with CD4^+^ T cells (57). In addition to producing antibodies, gingival B cells act as local antigen-presenting cells that sustain the expansion of T helper cells during chronic periodontitis (57, 58). Notably, under persistent pathogenic stimulation, locally activated B cells become a significant source of receptor activator of nuclear factor-κB ligand (RANKL), thereby linking chronic adaptive immune activation directly to osteoclastogenesis and alveolar bone resorption (5, 56).

Modern B-cell biology has also introduced refined subsets that are crucial for mucosal homeostasis. Transitional B cells represent a crucial developmental bridge between the bone marrow and peripheral lymphoid tissues, serving as a critical checkpoint for the elimination of autoreactive clones and the establishment of a functional peripheral B-cell repertoire (59). Within the tissue microenvironment, regulatory B cells (Bregs) act as essential controllers of inflammation, suppressing pathogenic T-cell responses and osteoclastogenesis primarily via the secretion of interleukin-10 (IL-10) and transforming growth factor beta (TGF-β) (55). Bregs contribute to periodontal immune homeostasis by limiting excessive Th1 and Th17 responses, a suppression primarily mediated through the secretion of IL-10 (56, 60). Conversely, double-negative (DN) B cells (CD19+CD27−IgD−) tend to accumulate during aging and chronic inflammatory conditions and have been associated with persistent immune activation (61).

Similarly, current evidence regarding B-cell dynamics highlights a clear hierarchy of proof. Human data primarily offer associative evidence connecting B-cell infiltration, altered antibody profiles, or RANKL expression to clinical disease severity. Conversely, definitive evidence regarding whether B-cell dysfunction directly causes dysbiosis—or is merely a secondary consequence of chronic mucosal inflammation—is largely restricted to experimental knock-out models or targeted depletion therapies. Maintaining this distinction between clinical correlation and preclinical causation remains essential for interpreting mucosal humoral immunity.

## 5 Mechanisms of microbiota-induced immune dysregulation and autoimmunity

Several oral microorganisms have been implicated in the initiation or amplification of autoimmune responses. One example is *P. gingivalis* and one of its main virulence factors, *Porphyromonas gingivalis* peptidylarginine deiminase (PPAD). A study conducted by Konig et al. aimed to determine the correlation between *P. gingivalis* PPAD and its effect on rheumatoid arthritis (RA). Therefore, they set out to analyze PPAD in *P. gingivalis* cells to detect citrullination. Subsequently, immunoglobulin G (IgG) antibodies against citrullinated recombinant PPAD (rPPAD) and unmodified rPPAD were measured in healthy individuals and those with RA. The study challenged the hypothesis that PPAD autocitrullination represents a major trigger of RA-associated autoimmunity (62). Maresz et al. also sought to demonstrate the correlation between these two diseases, while emphasizing the significant role of PPAD. The study was conducted using a mouse model with a titanium coil implanted subcutaneously. The mice were divided into several groups, each of which was analyzed, among other things, from immunological and histological perspectives (63). These findings complement rather than directly contradict those of Konig et al., as the two studies addressed different aspects of the relationship between *P. gingivalis* and RA (62). Citrullination generates neoepitopes that may contribute to the breakdown of immune tolerance and subsequent production of anti-citrullinated protein antibodies (ACPAs) (64).

Bystander activation of immune cells has also been proposed as a potential mechanism linking oral microbiota-associated inflammation with autoimmunity. In this context, *Porphyromonas gingivalis* may contribute to a pro-inflammatory environment through the release of outer membrane vesicles (OMVs), which carry key virulence factors including gingipains, lipopolysaccharide, and bacterial nucleic acids. *P. gingivalis* OMVs have been shown to deliver functional gingipains that degrade extracellular targets and stimulate host cells, driving the production of pro-inflammatory cytokines (such as IL-6 and IL-8) through MAPK, NF-κB, and STING-related pathways (65–67). Although these findings demonstrate potent inflammatory activity of *P. gingivalis* OMVs, their direct role in inducing bystander activation and autoimmunity remains to be established. In addition, *P. gingivalis* can manipulate the complement system through C5a receptor (C5aR) signaling. Its gingipains can generate biologically active C5a from C5, thereby activating C5aR and interfering with protective immune functions. Cross-talk between C5aR and TLR2 signaling can impair effective bacterial clearance while maintaining inflammation, thereby contributing to a chronic inflammatory environment and periodontal dysbiosis (68).

The bacteria responsible for periodontal inflammation are also capable of inducing epitope spreading. Citrullination of host proteins, induced by *P. gingivalis* and *A. actinomycetemcomitans*, leads to the broadening of the autoimmune response to subsequent antigens (69).

While *Porphyromonas gingivalis* has been proposed as a keystone pathogen capable of promoting polymicrobial dysbiosis, other periodontal-associated species contribute through distinct mechanisms of immune evasion, local inflammation, and systemic dissemination. For example, the major virulence factor of *Aggregatibacter actinomycetemcomitans*, leukotoxin (LtxA), selectively targets human leukocytes expressing β2 integrins (LFA-1), inducing pore formation, activation of the NLRP3 inflammasome, and cell death in neutrophils, monocytes, macrophages, and, under certain conditions, lymphocytes (70, 71). Furthermore, *A. actinomycetemcomitans* promotes the citrullination of host proteins through toxin-induced hypercitrullination, which triggers the production of autoantibodies and links localized periodontal destruction to systemic autoimmune reactivity, particularly rheumatoid arthritis (72). In contrast, *Fusobacterium nucleatum* facilitates physical aggregation between early commensals and late pathogenic colonizers, serving as a key bridging organism within the subgingival biofilm (73). From an immunological perspective, *F. nucleatum* uses outer membrane adhesins, including FadA and Fap2, to adhere to and penetrate host epithelial barriers (74). Fap2 directly interacts with TIGIT (T-cell immunoreceptor with Ig and ITIM domains) on natural killer (NK) cells and tumor-infiltrating lymphocytes, thereby suppressing cytotoxic immune responses (75). Furthermore, *F. nucleatum* can translocate across compromised epithelial barriers into the systemic circulation, contributing to systemic inflammation and being associated with colorectal carcinogenesis and adverse pregnancy outcomes (76, 77). *T. denticola* also plays a significant role in this context. This bacterium enhances tissue penetration and immune evasion through its Major Surface Protein (Msp) and dentilysin, a chymotrypsin-like protease complex (78). Dentilysin degrades extracellular matrix proteins, cleaves complement components such as C3, and modulates local inflammatory cytokine networks (79). This proteolytic activity disrupts immune cell recruitment and tissue barrier repair, promoting chronic tissue destruction and facilitating systemic inflammatory signaling within the vascular system.

## 6 Cross-talk between lymphocytes and the microbiome in disease and clinical contexts

### 6.1 T-cell–microbiome interactions in periodontal disease, candidiasis, and systemic conditions

Due to their broad functional repertoire, T lymphocytes may serve as indicators of changes occurring in the oral microbiome. An example is the study by Davanian et al., which included 41 patients with periodontitis (80). Additional clinical data regarding comorbid chronic diseases were collected. Inflamed periodontal tissues were excised and analyzed for T cell receptor (TCR) repertoires, alongside microbiological profiling. The dominant bacterial phyla identified were *Proteobacteria*, *Bacteroidetes, Firmicutes*, and *Fusobacteria.* The authors highlighted the potential involvement of MAIT cells in periodontal inflammation. The TCR repertoire analysis suggested the presence of MAIT-like populations, characterized by semi-invariant TCRα chain usage (TRAV1-2–TRAJ33, also referred to as Vα7.2–Jα33). This indicates that the oral microbiome may modulate local immune responses, at least in part, through MAIT cell activation and expansion (80).

In the context of microbiome–lymphocyte interactions, *Candida albicans* represents an important oral fungal commensal that can become an opportunistic pathogen when mucosal immune or epithelial barrier homeostasis is disrupted. STAT3-dependent Th17 immunity is critical for mucosal antifungal defense against *C. albicans*. In humans, STAT3 deficiency impairs Th17 differentiation and is associated with susceptibility to mucocutaneous candidiasis and alterations in the oral microbial community (81). Experimental studies further indicate that IL-22–STAT3 signaling in oral epithelial cells contributes to antifungal barrier defense, complementing IL-17-dependent immune responses during oral candidiasis. Together, these findings illustrate how defects in adaptive mucosal immunity can alter the oral microbial ecosystem and support the broader concept of bidirectional interactions among the oral microbiota, lymphocytes, and the epithelial barrier (82).

Milner et al. demonstrated that loss-of-function mutations in STAT3 are associated with impaired Th17 cell development, resulting in increased susceptibility to chronic mucocutaneous candidiasis (83). Another key component of antifungal immunity is interleukin-17 (IL-17), produced predominantly by Th17 cells. IL-17 plays a central role in granulopoiesis and neutrophil recruitment, thereby facilitating pathogen clearance and protecting the host against candidal infections (82). Studies by Hernández-Santos et al. in both murine models and humans showed that deficiency of the IL-17 receptor (IL-17R) leads to chronic mucocutaneous candidiasis, highlighting the essential role of IL-17–mediated signaling in antifungal immunity (32).

An important link between the oral microbiome and T lymphocyte responses was demonstrated in a study by Tsukasaki et al. The experiments were performed in multiple murine models, including wild-type mice, mice deficient in IL-17 signaling, mice lacking the interleukin-6 receptor (IL-6R), mice with conditional deletion of IL-6R in regulatory T cells, and mice treated with IL-6R-blocking antibodies, alongside appropriate control groups (5). Inflammation and bone resorption were experimentally induced, followed by immunophenotyping of T cell subsets. Subsequently, antibiotic treatment was administered to assess changes in the oral microbiota. The results indicated that Th17 cells play a central role in the immune response to oral bacterial communities. Their function is associated with modulation of microbiome composition and promotion of inflammatory responses that contribute to osteoclast activation and bone resorption. These findings suggest that Th17-driven inflammation, while contributing to host defense against bacterial invasion, is also closely linked to pathological bone loss in periodontitis (5).

Kou et al. demonstrated a significant association between Th17 and Treg lymphocytes and the oral microbiome in the context of periodontal inflammation (6). The study aimed to investigate the roles of Th17 and Treg cells in periodontal disease progression. Experimental periodontitis was first induced, followed by *Porphyromonas gingivalis* lipopolysaccharide (Pg-LPS) administration. Inflammatory mediators were quantified using an enzyme-linked immunosorbent assay (ELISA), and alveolar bone loss was also assessed. In addition, the proportions of Th17 and Treg cells were analyzed, and immunofluorescence techniques were applied. The results revealed a disruption in the Th17/Treg balance, characterized by an increase in pro-inflammatory Th17 responses and a relative impairment of Treg-mediated immunosuppression. This suggests that microbial stimulation in periodontal disease is associated with immune dysregulation rather than a functional conversion of Treg cells toward a pro-inflammatory phenotype, contributing to sustained local inflammation (6).

Taken together, these studies support a link between oral microbial dysbiosis and T-cell-mediated inflammation, particularly involving Th17-related pathways. However, their findings should be compared with caution, as Davanian et al. analyzed human periodontal tissues, whereas Tsukasaki et al. and Kou et al. relied mainly on experimental murine models. Human studies provide greater clinical relevance but are affected by variability between individuals, while animal models allow more controlled mechanistic assessment but may not fully represent the complexity of human periodontal disease (5, 6, 80).

The relationship between the immune system, including T lymphocytes, and the oral microbiome is also relevant in the context of pregnancy. Yang et al. conducted a study involving 26 pregnant women, from whom samples were collected from the oral cavity and peripheral venous blood, among other sites. High-throughput sequencing was used to characterize the oral microbiota, while flow cytometry was applied to assess immune cell populations. The results demonstrated significant associations between the composition of the oral microbiome and T lymphocyte subsets, including CD4^+^ T cells, as well as the CD4^+^/CD8^+^ ratio. Although the study did not identify specific causative bacterial taxa responsible for these correlations, several genera were highlighted, including *Prevotella* spp., *Corynebacterium* spp., and *Bacteroides* spp. (84).

The oral microbiome may also influence immune responses in patients with metabolic diseases. This was investigated in a study by He et al., which first assessed periodontal status in participants and subsequently analyzed saliva samples using 16S ribosomal RNA (16S rRNA) gene sequencing (85). In a second phase, diabetic mouse models were established and subjected to microbiota transplantation, in which animals received saliva from either periodontally healthy individuals or patients with periodontitis, followed by assessment of immune responses. The results suggested that oral microbiome dysbiosis associated with periodontitis may contribute to altered immune responses in the context of diabetes, including enhanced Th17-mediated inflammation. The bacterial taxa implicated in this dysbiosis included *Porphyromonas gingivalis*, *Treponema denticola*, and *Fusobacterium nucleatum* (85).

### 6.2 T-cell dysregulation in oral lichen planus

Oral lichen planus (OLP) is a chronic T-cell-mediated inflammatory disease of the oral mucosa, which offers a clinically relevant model of dysregulated adaptive mucosal immunity. Antigen-specific pathways include antigen presentation by basal keratinocytes and their subsequent destruction by cytotoxic CD8^+^ T lymphocytes, contributing to basement membrane disintegration and keratinocyte apoptosis (38). More recent single-cell profiling of OLP lesions identified activated tissue-resident memory CD8^+^ T cells and clonal expansion of the T-cell receptor repertoire, supporting sustained local antigen-driven immune activation (86). Regulatory mechanisms are also altered: tissue studies have demonstrated increased forkhead box P3 (FOXP3)-positive regulatory T cells, whereas findings regarding interleukin-17 (IL-17)-producing cells have been less consistent, indicating a complex and context-dependent relationship between T helper 17 (Th17) and regulatory T (Treg) cells (87). Mucosal-associated invariant T (MAIT) cell phenotypes have also been identified in the connective tissue of OLP lesions, and their abundance differed among groups classified according to symptoms and the presence of *Candida*, suggesting a potential relationship between microbially responsive lymphocytes and mucosal inflammation (88). In parallel, microbiome analyses have identified differences in salivary and lesional bacterial communities between patients with OLP and healthy controls, as well as bacterial signals within OLP tissues (89). However, these cross-sectional findings do not establish whether microbial dysbiosis initiates T-cell activation or develops as a consequence of chronic mucosal inflammation.

### 6.3 B-cell dynamics in aging, periodontitis, and depletion therapy

Aging may affect B lymphocyte function. With increasing age, an elevated frequency of senescence-associated B cells has been observed in peripheral blood (90). These cells exhibit a senescence-like phenotype characterized by loss of proliferative capacity, while maintaining transcriptional and metabolic activity and producing elevated levels of pro-inflammatory cytokines, thereby contributing to remodeling of the tissue microenvironment and modulation of immune responses. Although these cells do not proliferate, they may still participate in immune regulation; however, their functional capacity is altered. Senescence-associated B cells have been implicated in impaired humoral immune responses and in the development of chronic low-grade inflammation observed in age-related conditions such as type 2 diabetes and inflammatory bowel disease. Collectively, these findings suggest that aging contributes to the accumulation of non-proliferative but metabolically active B cell subsets, which may impair effective antibody responses and promote a persistent pro-inflammatory state (91).

Another study investigated B cell function in periodontitis using a murine model. The results showed that experimental periodontitis, associated with oral microbial dysbiosis, leads to increased B cell accumulation in the salivary glands, along with enhanced production of immunoglobulin A (IgA) (54). These findings suggest that dysbiosis of the oral microbiota can stimulate mucosal immune responses, promoting recruitment or local expansion of B cells and increased IgA secretion, which contributes to the maintenance of mucosal barrier immunity. This indicates that B cells are capable of rapidly responding to alterations in the oral microbial environment by enhancing local antibody-mediated defense mechanisms (92).

The aging process may contribute to alterations in the relationship between B lymphocytes and the microbiota. An example supporting this hypothesis is a study conducted by Mizuno et al., in which multiple murine groups were maintained under different conditions, including germ-free environments, microbiome-modified settings, and models with altered B cell function. Microbial composition was assessed using 16S rRNA gene sequencing (93). The results demonstrated age-associated shifts in microbial composition, including an increase in *Streptococcus* spp. and a decrease *in Staphylococcus* spp. These changes were correlated with alterations in B cell–derived immunoglobulin A (IgA) levels, with reduced IgA associated with greater microbial dysbiosis. These findings suggest that age-related declines in IgA-mediated mucosal immunity may contribute to changes in microbial homeostasis. However, extrapolation to specific oral diseases, such as dental caries associated with *Streptococcus mutans*, should be interpreted with caution, as disease development is multifactorial and not solely determined by IgA levels (94, 95).

The impact of B lymphocytes on the oral microbiome was also investigated by Troci et al. The study included three groups: patients with multiple sclerosis who had initiated B cell–depleting therapy, patients who had not received such treatment, and healthy controls. In all groups, the oral and intestinal microbiota were analyzed using 16S rDNA sequencing. In addition to identifying differences in microbial composition between individuals with multiple sclerosis and healthy controls, the study observed that B cell–depleting therapy was associated with shifts in oral bacterial communities, including increased diversity of *Streptococcus* and *Actinomyces* spp., alongside a reduction in *Porphyromonas* and *Veillonella* spp. (96). These taxa have been previously implicated in periodontal dysbiosis; however, their roles are context-dependent and involve complex interactions within the oral microbial ecosystem (97, 98).

The available evidence regarding B cells is more heterogeneous and often indirect. Studies in animal models provide mechanistic insight into IgA-mediated mucosal regulation, whereas clinical observations, such as those involving B-cell-depleting therapy, suggest associations between altered B-cell function and shifts in oral microbial composition. However, disparities in clinical context, patient immune status, and microbiome sampling methodologies restrict direct comparisons, leaving it difficult to definitively resolve whether B-cell reprogramming is an upstream driver or a downstream consequence of microbial dysbiosis.

## 7 Tables and comparative summaries

A summary of the above information is provided in **Table 1**.

**Table 1:** Comparison of the influence of B and T lymphocytes on the oral microbiome.

Type of immune system cell	Source	Interacting pathways / molecular signals	Impact on the oral microbiome	Other activities
T lymphocytes	(5, 6, 32, 36, 80, 84)	-> STAT3 pathway; -> IL-6 / IL-6R signaling; -> Wnt signaling; ->CD103 – E-cadherin interaction; ->IL-17 / IL-17R signaling	->STAT3/IL-17 axes protect against *C. albicans* overgrowth; -> Th17-driven responses modulate microbiome composition; -> Positive correlation with *Prevotella*, *Corynebacterium*, and *Bacteroides*.	-> IL-6R and Th17 pathways promote osteoclast activation and bone resorption in periodontitis; -> Wnt signaling restrains CD8+ terminal differentiation; ->Treg phenotype shifts toward Th17-like in periodontitis;
B lymphocytes	(53; 99–101)	->BCR signaling cascade; ->CD40–CD40L / ICOS–ICOSL / CD28–CD86 costimulation; -> IL-10-mediated suppression; -> RANK – RANKL pathway; -> C5aR (Complement) crosstalk;	-> Reduced B cell activity is associated with an increase in *Streptococcus* and *Actinomyces* and a decrease in *Staphylococcus*, *Porphyromonas*, and *Veillonella*.	-> Chronically activated B cells drive alveolar bone destruction via RANKL expression; -> IL-10 production by regulatory B cells attenuates Th1-driven inflammation; -> Mutations in pre-BCR/downstream cascades lead to immunodeficiency;

A narrative summary of the main oral microorganism–lymphocyte interactions discussed in this review is presented in **Table 2**.

**Table 2:** Narrative summary of the main oral microorganism–lymphocyte interactions discussed in this review.

Oral microorganism / microbial context	Relevant lymphocyte subset(s)	Key immune mediators / mechanisms	Associated oral condition or relevance	Evidence basis	Sources cited in the manuscript
*Candida albicans*	Th17 cells	STAT3-dependent Th17 differentiation, IL-17 signaling, and neutrophil recruitment	Oral candidiasis and mucosal immune defense	mechanistic and clinical observations.	(32, 82, 83)
Periodontal dysbiotic biofilms, including *Porphyromonas gingivalis*	Th17/Treg axis	IL-17-mediated inflammation, reduced regulatory control, osteoclast activation, and alveolar bone resorption	Periodontitis and periodontal tissue destruction	experimental periodontitis models and human periodontal observations.	(5, 6)
*Porphyromonas gingivalis* and Pg-LPS	Th17 and Treg cells	Th17 expansion, Th17/Treg imbalance, inflammatory mediator production, and impaired immunosuppression	Periodontal inflammation	experimental models.	(6)
Mixed periodontal communities including *Treponema denticola* and *Fusobacterium nucleatum*	Th17-related responses	Dysbiosis-associated immune activation and enhanced inflammatory responses	Periodontitis, including diabetes-associate periodontal inflammation	clinical and experimental observations.	(85)
Oral bacterial communities in inflamed periodontal tissues	MAIT-like T-cell populations	Semi-invariant TCR usage and microbial antigen-driven local T-cell activation	Periodontal inflammation	human observational evidence.	(80)
Commensal oral biofilms	Treg cells	Immune tolerance and suppression of excessive inflammatory responses	Oral immune-microbial homeostasis	biologically plausible mechanism.	(6, 9)
Dysbiotic oral microbiota	B cells and plasma cells	IgA production, mucosal antibody response, and microbial immune exclusion	Mucosal barrier defense and periodontitis-associated dysbiosis	animal and observational findings.	(92, 93)
Chronic periodontal pathogens	B cells	Chronic antigenic stimulation, RANKL expression, osteoclastogenesis, and inflammatory B-cell activity	Alveolar bone loss and periodontal tissue destruction	experimental evidence.	(5)
Altered oral communities including *Streptococcus*, *Actinomyces*, *Porphyromonas*, and *Veillonella*	B-cell-dependent responses	B-cell depletion-associated shifts in oral bacterial composition	Oral microbial dysbiosis in systemic disease contexts	associative clinical observations.	(96–98)
Oral dysbiotic biofilms	Regulatory B cells (Bregs)	IL-10-mediated suppression of Th1/Th17 responses and limitation of excessive inflammation	Periodontal immune regulation and oral immune-microbial balance	mechanistic studies.	(55, 56, 60)

## 8 Discussion

This narrative review highlights the complex network of interactions between components of adaptive immunity and the oral microbiome. The analyzed evidence indicates that T and B lymphocytes are not passive bystanders of microbial colonization, but active regulators that contribute to the balance between immune homeostasis and dysbiosis (**Figure 1**). For instance, Th17 cells exhibit context-dependent functions: they are essential for antifungal immunity and control of *Candida* species, yet their excessive or dysregulated activation in periodontal inflammation, partly driven by biofilm-associated signals, contributes to tissue damage and bone resorption. B lymphocytes also play a multifaceted role that extends beyond secretory IgA (sIgA) production. Although sIgA contributes to immune exclusion and the control of cariogenic and commensal bacteria, B cells additionally function as antigen-presenting cells and immunoregulatory cytokine producers. Moreover, evidence from B cell–depletion studies suggests that modulation of B cell activity may lead to shifts in microbial community composition in certain disease contexts, indicating an indirect role in maintaining or disrupting microbial homeostasis. Importantly, the relationship is bidirectional, as microbial communities actively shape lymphocyte differentiation, activation, and functional polarization. In selected pathological settings, particularly periodontitis-associated dysbiosis, interactions between oral microorganisms and lymphocyte responses may contribute to systemic immune dysregulation through mechanisms such as protein citrullination and molecular mimicry. However, such findings should be interpreted cautiously, as they may reflect broader immune rebalancing rather than a direct “resetting” of the microbiome.

**Figure 1:**
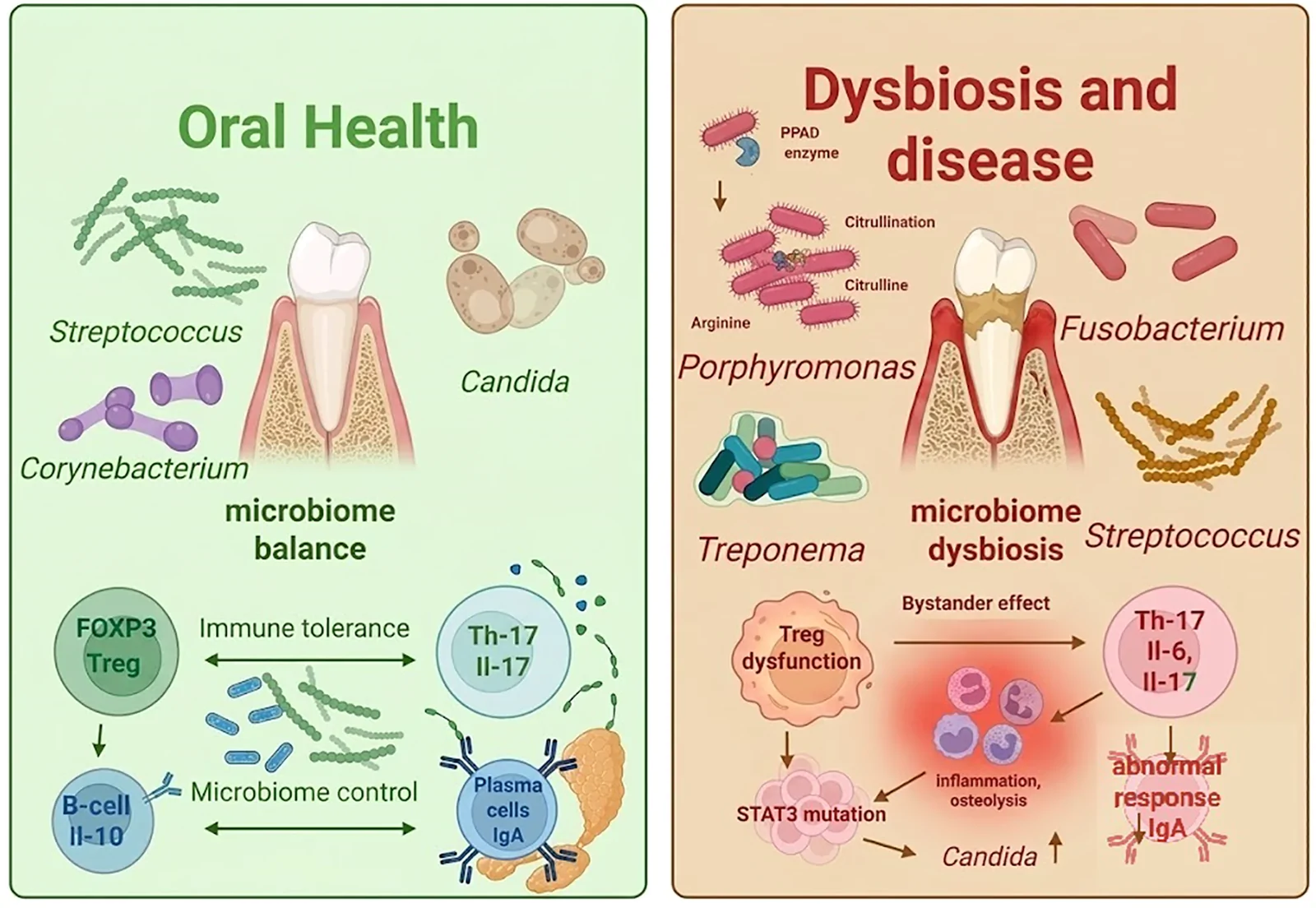
The role of T and B cells in maintaining a healthy oral microbiome. Illustration created using BioRender.com.

Taken together, the evidence synthesized across conventional T- and B-cell subsets, innate-like lymphocytes, and systemic inflammatory pathways supports a complex, highly coordinated network interacting with the oral microbiome. However, a critical synthesis of these findings requires maintaining a clear distinction between levels of evidence: clinical human studies mainly reveal strong correlations, whereas definitive mechanistic causality is largely derived from experimental animal and *in vitro* models. Furthermore, methodological variations in sampling sites, disease stages, and analytical platforms limit direct comparisons across studies, leaving the primary causal direction between lymphocyte dysregulation and microbial dysbiosis unresolved. Human studies offer clinical relevance, whereas animal and *in vitro* models provide stronger mechanistic insight but limited translational certainty. Therefore, the available evidence suggests a biologically important interaction between adaptive immunity and oral microbial communities, although its causal direction and clinical implications require further investigation. Several questions therefore remain unresolved, including whether lymphocyte dysregulation primarily drives oral microbial dysbiosis or whether dysbiotic microbial communities initiate and shape lymphocyte activation. Future studies should prioritize longitudinal human research integrating high-resolution oral microbiome profiling with deep immune-cell phenotyping and standardized, site-specific sampling protocols.

## 9 Conclusions and future directions

In summary, oral homeostasis is not a static state but the result of continuous and dynamic immune–microbial interactions, in which T and B lymphocytes act as key modulators of the oral microbial ecosystem. The available evidence suggests that maintenance of tissue integrity depends on the balanced regulation of immune cell subsets, particularly the Treg/Th17 axis and the effector and regulatory functions of B lymphocytes. Dysregulation of these pathways is associated with the development and persistence of periodontal inflammation and with ecological shifts that favor pathogenic biofilm formation. Recognition of these interactions supports a potential shift from exclusively antimicrobial approaches in dentistry toward strategies that also consider host immune modulation and restoration of mucosal immune balance. Future research should integrate high-throughput metagenomic approaches with single-cell transcriptomic analyses to move beyond correlative associations and better define the relationships between specific microbial antigens and lymphocyte differentiation pathways. The incorporation of multi-omics approaches, including metabolomics and analyses of immunosenescence, may further contribute to a more comprehensive understanding of oral immune–microbiome interactions and support the development of more individualized diagnostic and therapeutic strategies in precision dentistry. Crucially, while evidence from preclinical models confirms that specific immune cellular pathways can actively drive or restrain dysbiosis, translational application in humans must respect that clinical observations currently reflect association rather than proven causality.
